# Retinoic Acid Increases Proliferation of Human Osteoclast Progenitors and Inhibits RANKL-Stimulated Osteoclast Differentiation by Suppressing RANK

**DOI:** 10.1371/journal.pone.0013305

**Published:** 2010-10-11

**Authors:** Lijuan Hu, Thomas Lind, Anders Sundqvist, Annica Jacobson, Håkan Melhus

**Affiliations:** Department of Medical Sciences, Uppsala University, Uppsala, Sweden; Texas A&M University, United States of America

## Abstract

It has been shown that high vitamin A intake is associated with bone fragility and fractures in both animals and humans. However, the mechanism by which vitamin A affects bones is unclear. In the present study, the direct effects of retinoic acid (RA) on human and murine osteoclastogenesis were evaluated using cultured peripheral blood CD14^+^ monocytes and RAW264.7 cells. Both the activity of the osteoclast marker tartrate resistant acid phosphatase (TRAP) in culture supernatant and the expression of the genes involved in osteoclast differentiation together with bone resorption were measured. To our knowledge, this is the first time that the effects of RA on human osteoclast progenitors and mature osteoclasts have been studied *in vitro*. RA stimulated proliferation of osteoclast progenitors both from humans and mice. In contrast, RA inhibited differentiation of the receptor activator of nuclear factor κB ligand (RANKL)-induced osteoclastogenesis of human and murine osteoclast progenitors via retinoic acid receptors (RARs). We also show that the mRNA levels of receptor activator of nuclear factor κB (RANK), the key initiating factor and osteoclast associated receptor for RANKL, were potently suppressed by RA in osteoclast progenitors. More importantly, RA abolished the RANK protein in osteoclast progenitors. This inhibition could be partially reversed by a RAR pan-antagonist. Furthermore, RA treatment suppressed the expression of the transcription factor nuclear factor of activated T-cells cytoplasmic 1 (NFATc1) and increased the expression of interferon regulatory factor-8 (IRF-8) in osteoclast progenitors via RARs. Also, RA demonstrated differential effects depending on the material supporting the cell culture. RA did not affect TRAP activity in the culture supernatant in the bone slice culture system, but inhibited the release of TRAP activity if cells were cultured on plastic. In conclusion, our results suggest that retinoic acid increases proliferation of human osteoclast progenitors and that it inhibits RANK-stimulated osteoclast differentiation by suppressing RANK.

## Introduction

Vitamin A, including its active metabolite retinoic acid (RA), is required for normal growth and is a potent regulator of physiological activities such as vision, reproduction, growth and development in vertebrates [1]. In particular its roles in the skeletal development and metabolism have been recognized since a long time [2]. Hypervitaminosis A causes accelerated thinning of long bones, bone fragility and spontaneous fractures in animal experiments. Furthermore, high intake of dietary vitamin A and serum retinol levels have been associated with increased risk of hip fractures in humans [3], [4], [5], [6]. Retinoic acid is suggested to mediate much of the physiological effects of retinol via binding to retinoic acid receptors (RARα, β, γ) which form heterodimers with retinoic X receptors (RXRα, β, γ) [7]. The RAR/RXR heterodimers function as transcription factors, activating specific RA response elements (RAREs) for transcriptional regulation of the target genes [7], [8].

Osteoclasts are multinucleated, TRAP positive and bone resorbing giant cells derived from hematopoietic cells of the myeloid lineage. Macrophage colony-stimulating factor (M-CSF, also termed CSF1) and RANKL (Tnfsf11) are essential molecules for the differentiation of osteoclasts, and are abundantly expressed in stromal cells/osteoblasts [9], [10], [11]. M-CSF supports proliferation and survival of osteoclast precursors and increases the expression of the receptor for RANKL, i.e. RANK (Tnfrsf11a) [12], [13]. The interaction between RANKL and RANK is critical for osteoclast differentiation and activation of mature osteoclasts [9], [10], [11]. Mice deficient in M-CSF, RANKL, or RANK have defect osteoclasts and develop osteopetrosis [14], [15], [16], [17]. Stromal cells/osteoblasts release osteoprotegerin (OPG) which is a soluble decoy receptor for RANKL and functions as a potent inhibitor of osteoclastogenesis. The rate of osteoclastogenesis and bone resorption under physiological conditions of bone homeostasis is correlated to the ratio of RANKL to OPG. A targeted deletion of OPG in mice often results in multiple fractures, numerous osteoclasts, and decreased trabecular bone volume [18].

RANKL signals through RANK in osteoclast precursors and sequentially activates nuclear factor kappa-light-chain-enhancer of activated B cells (NF-κB) and mitogen-activated protein kinases (MAPKs). RANK signaling additionally leads to induction of c-Fos, which is a prerequisite condition for the induction of NFATc1, a master transcription factor for osteoclastogenesis. MafB and IRF-8 are transcription factors that are abundantly expressed in osteoclast precursors and are thought to be important negative regulators of osteoclast differentiation [19], [20]. MafB and IRF-8 suppress the activity of NFATc1 and thereby reduce the expression of NFATc1 target genes. When RANK is activated by RANKL, it sends signals into TNF receptor-associated factor molecules (TRAFs), leading to up-regulation of the downstream molecules such as c-Fos and down-regulation of MafB and IFR-8 which regulate NFATc1 activity and its auto-amplification, resulting in the activation, survival and differentiation of osteoclasts.

The effects of retinoids on osteoclasts have been examined *in vitro*, but findings vary between different systems. Studies of organ cultured bone consistently show increased resorption. Similarly, the overwhelming amount of data in mature chicken and rabbit osteoclasts show increased activity by RA [21], [22], [23], [24], [25], [26]. In contrast, the majority of data show that RA inhibits osteoclast differentiation when cells from murine bone marrow have been used. In *vivo* studies suggest that RA has opposite affects on trabecular and cortical bone [25]. The effects on human osteoclast progenitors have not been studied. Since RA has effects on both human osteoblasts [27] and stromal cells [28], a pure cell culture system is required to evaluate the direct molecular mechanisms. In the present study, we therefore investigated the effects of RA on osteoclast progenitors from purified peripheral blood CD14^+^ monocytes. The effects of retinoids may be influenced not only by the culture system and sources of osteoclasts, but also by species [21], [25], [29], [30], [31], [32], [33]. For this reason we studied the effects of RA on both human and murine osteoclast progenitors and on RANKL-induced maturation of these cells, cultured on bone as well as plastic substrates.

## Materials and Methods

### Materials

Recombinant human M-CSF and RANKL were obtained from Nordic BioSite AB (Sweden). Bone slices and Cross Laps ELISA kits were from Immunodiagnostic Systems Nordic (Denmark). All-*trans*-retinoic acid (RA) was purchased from Sigma Aldrich, Sweden and was dissolved in 95% ethanol in the dark room under the flow of nitrogen. The stock solution (0.5 mg/ml or 1.66 mM) was stored at −70°C and shielded from light until use. The RAR pan-antagonist AGN194310 (AGN), which was dissolved in dimethyl sulfoxide (DMSO), was kindly provided by Dr. Chandraratna, Allergan Inc, Irvine, CA USA.

### Osteoclast formation in RAW264.7 cell culture

RAW264.7 cells (from ATCC) were cultured as described previously [34]. In brief, the cells were seeded in a 96-well plate either on plastic or on bone slices at a density of 1.5×10^3^ cells per well. Cells were cultured for 6 or 7 days in the control medium (α-MEM containing 10% FBS, 2 mM L-glutamine and 1% penicillin) or in the control medium in the presence of 100 ng/ml recombinant human RANKL with or without RA and AGN.

### Osteoclast formation in the human CD14^+^ cell culture

Isolation and differentiation of CD14^+^ monocytes were carried out as described previously [35]. In brief, buffy coat blood was obtained from anonymous healthy donors at Uppsala University Hospital. Uppsala University Hospital ethics committee approved the study and waived the need for consent from donors. The peripheral blood mononuclear cells (PBMCs) were isolated by Ficoll-Paque Plus (GE healthcare, Sweden) density gradient centrifugation according to the manufacturer's instruction. The PBMCs were re-suspended in cold PBS containing 0.5% BSA and 2 mM EDTA. The human CD14^+^ monocytes were isolated using the positive immunomagnetic selection based on the cell surface markers by human CD14^+^ beads from Miltenyi Biotec (Germany). Human CD14^+^ cells were seeded in the control medium with 25 ng/ml M-CSF for 3 days. By the third day, the cells were detached by incubation with trypsin at 37°C for 20 minutes and removed by a scraper. Then the cells were seeded in a 96-well plate at a density of 1.5×10^5^ cells per well on bone slices or 5×10^4^ cells on plastic with the control medium supplemented with M-CSF and RANKL each at 25 ng/ml as previously described [35]. For the osteoclast progenitor experiment, 4 nM of RA and 8 nM of the AGN were added on day 0 and incubated for 3 days or 2 weeks. For the mature osteoclast experiment RA was added on day 14 for 2, 8 or 12 days. The culture medium was replaced every second day during the experiment.

### The proliferation of osteoclast progenitors

RAW264.7 cells were cultured in the absence or presence of 100 ng/ml recombinant human RANKL with or without RA in a 96-well plate for 6 days. Human CD14^+^ cells were seeded in the control medium with 25 ng/ml of M-CSF in the absence or presence of 25 ng/ml of RANKL with or without 4 nM of RA in a 96-well plate or 12-well plate for 14 days. The cell proliferation was measured with the MTT kit (Sigma Aldrich, Sweden) in a 96-well plate or by cell number counting in a 12-well plate with NucleoCounterTM (Chemometec, Allerød, Denmark) following the manufacturer's instructions.

### Determination of Osteoclast Formation

Osteoclasts were identified by measuring TRAP activity in the culture medium. A more than 3-fold increase of the TRAP activity compared with control indicates the formation of osteoclasts. The TRAP activity measurement was carried out using an adapted Sigma protocol as described [36]. Briefly, medium was added to the ELISA plates containing 0.1 M acetate (pH 5.2), 0.15 M potassium chloride, 0.1% triton X-100, 1 mM ascorbic acid Na-salt, 0.1 mM ammonium ferrious sulfate hexahydrate, 10 mM phosphatase substrate *p*-nitrophenyl phosphate (PNPP) and 10 mM Na-tartrate acid buffer. The plate was incubated at 37°C for 30 minutes. The reaction was stopped with the addition of 0.3 N NaOH and absorbance was measured at 405 nm. TRAP catalyzes the conversion of PNPP to *p*-nitrophenol (PNP), giving a maximal absorbance at 405 nm, which corresponds to the TRAP activity in the sample. Also, TRAP^+^ cells containing 3 or more nuclei were considered as osteoclasts. The TRAP staining was carried out using the Acid Phosphatase, Leukocyte (TRAP) Kit (Sigma-Aldrich Sweden).

### Resorption assay

The release of C-terminal telopeptide of type I collagen (CTX) from mineralized bone slices was determined using CrossLaps for Culture kit (Nordic Bioscience Diagnostics, Denmark) according to the manufacturer's instructions.

### RNA isolation and gene expression measured by Real-Time PCR

Human CD14^+^ cells were seeded in a 96-well plate at a density of 1.5×10^5^ cells or 5×10^4^ cells per well on bone slices or plastic, respectively, with the control medium supplemented with M-CSF and RANKL each at 25 ng/ml as previously described [35]. For the effects of RA in the osteoclast progenitor experiment, 4 nM of RA and 8 nM of AGN were added on day 0 for 3 days. On the third day the cells were washed twice with PBS and lysed in RLT lysis buffer (Qiagen). Total RNA was isolated using the RNeasy Kit (Qiagen). A one-step reaction was performed in a GeneAmp PCR System 9700 (Applied Biosystems) to reversely transcribe about 50 ng of total RNA into cDNA with the High Capacity cDNA reverse transcription kit (Applied Biosystems). The cDNA was then amplified using the TaqMan® real-time PCR on ABI PRISM® 7000 Sequence Detection Systems (Applied Biosystems). All reactions were performed in 96-well plates with a final volume of 25 µl per well. Cycling conditions were 2 minutes at 50°C followed by 10 minutes at 95°C to activate the Taq DNA Polymerase, and 40 cycles of 15 seconds at 95°C, and finally 60 seconds at 60°C. Quantitative PCR was performed using inventoried TaqMan® Gene Expression Assays for CD14, RANK, IRF-8, NFATc1 and cathepsin K mRNA measurements. The target gene expressions were compared with that of β-actin to obtain the relative mRNA expression.

### Western blot analysis

Human CD14^+^ cells were seeded at a density of 1×10^5^ cells/cm^2^ in a 24-well plate and incubated in the presence of 25 ng/ml of M-CSF and 25 ng/ml of RANKL with or without 4 nM of RA or 8 nM of AGN for 3 days. Cells were lysed and proteins were separated on an 8% SDS-PAGE gel and blotted onto a nitrocellulose membrane (GE healthcare, Sweden). The membrane was rinsed and blocked with 5% BSA in TBS/0.05% Tween-20 and incubated with RANK and β-actin antibodies (Santa Cruz biotechnology, CA.USA) for overnight at 4°C, followed by incubation with a horseradish-conjugated secondary antibody for 1 hour. The protein bands were visualized with an ECL detection system (GE healthcare, Sweden).

## Results

### RA inhibits osteoclast differentiation

**Figure 1:**
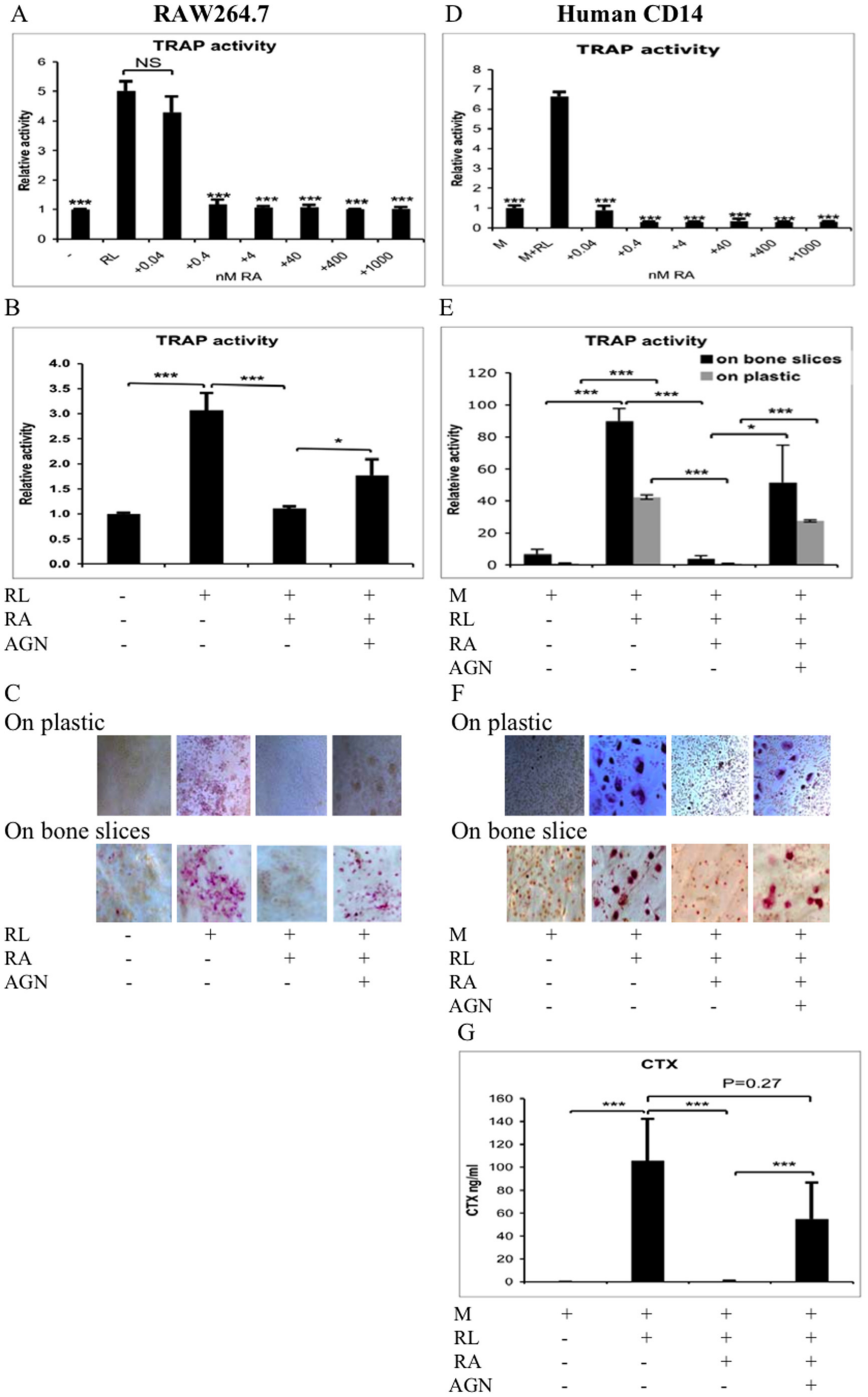
RA inhibits differentiation of osteoclast progenitors. TRAP activity in medium in RAW264.7 cells incubated with RANKL (RL, 100 ng/ml) with various concentrations of RA (A), or in the presence or absence of 4 nM of RA or 8 nM of RAR pan-antagonist (AGN) (B, C) on bone slices or plastic for 7 days was measured as described in materials and methods. Human CD14^+^ blood monocytes were incubated with M-CSF (M, 25 ng/ml), RANKL (RL, 25 ng/ml) and various concentrations of RA (D), or in the presence or absence of 4 nM RA or 8 nM AGN on bone slices for 14 days or on plastic for 10 days (E–G). Release of TRAP activity in the culture medium was determined using an adapted Sigma protocol (A, B, D, E). The TRAP staining was carried out using the Acid Phosphatase Leukocyte (TRAP) kit (C, F). CTX was determined by CrossLaps ELISA kit (G). Each data point represents the average ± SD of triplicate wells. Similar results were obtained in more than three independent experiments. NS means non-significant difference. A, D, compared with RL group. * P<0.05, *** P<0.001.

Secreted TRAP activity, a marker of the osteoclast resorption capacity [37], in the medium of murine RAW264.7 cells was significantly increased 3 to 5 times compared to cells not receiving RANKL on plastic (Figure 1A, B). TRAP staining of these cells revealed an abundance of osteoclasts with more than 3 nuclei both on plastic and bone slices (Figure 1C, expanded figure in Figure S2). However, no functional osteoclasts were formed, measured as CTX fragment generation, when the RAW264.7 cells were cultured with RANKL on bone slices (data not shown). RA inhibited the RANKL-induced osteoclast differentiation when it was added to the RAW 264.7 cell cultures on plastic (Figure 1A-C). RA at a concentration as low as 0.4 nM was sufficient to inhibit TRAP release in RAW 264.7 cells (Figure 1A). No multiple-nuclear TRAP positive cells were found in the presence of 4 nM RA (Figure 1C). The RAR pan-antagonist (AGN) partially reversed the inhibition of osteoclastic TRAP release and multi-nuclear TRAP-positive osteoclast formation by RA (Figure 1B, C). To test the effect of RA on human cells we used a standard, validated culture system for human osteoclastogenesis involving culturing peripheral blood purified CD14^+^ cells with M-CSF and RANKL for 10–14 days [35]. In these cultures TRAP-release was increased 42- and 13-fold compared with the cells not receiving RANKL seeded on plastic or bone slices, respectively (Figure 1E). Importantly, these cells were functional osteoclasts as the bone resorption activity was highly induced by RANKL, measured as CTX fragment generation, when cells were cultured on bone slices (Figure 1G). RA inhibited the RANKL-induced osteoclast differentiation when it was added to human CD14^+^ cells both on plastic and bone slices (Figure 1D–F). The inhibition can even be observed at a concentration as low as 0.04 nM of RA on human CD14^+^ cells (Figure 1D and Figure S1). RA at 4 nM in the human CD14^+^ cell culture in the presence of M-CSF and RANKL almost completely abolished the formation of multi-nuclear TRAP-positive cells together with a near total elimination of TRAP- and CTX-release (Figure 1D–G, Figure 1F was expanded in Figure S2). Osteoclast formation, TRAP- and CTX-release were largely restored in the presence of the RAR-pan-antagonist (Figure 1E–G). These results were consistent irrespective of the carrier for the cell culture, i.e. plastic or bone.

### RA increases osteoclast progenitor proliferation

**Figure 2:**
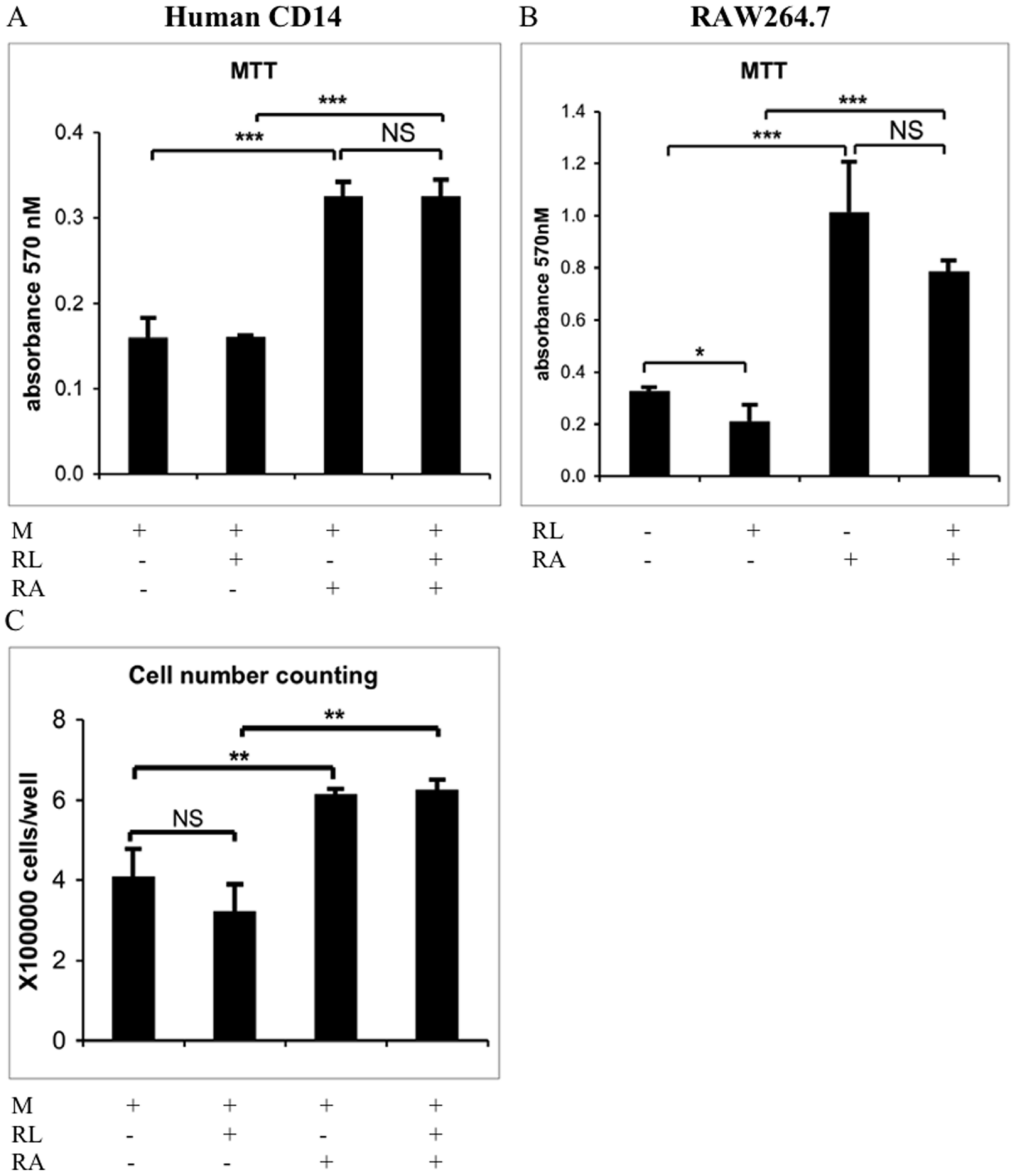
RA increases proliferation of the osteoclast progenitors. RAW264.7 cells (A) were incubated in a 96-well plate in the presence or absence of RANKL (RL) and 4 nM of RA for 6 days. Human CD14^+^ cells were incubated in a 96-well plate (B) or 12-well plate (C) in the absence or presence of M-CSF (M), RANKL (RL), and 4 nM of RA for 14 days. Cell proliferation was determined by an MTT based cell growth determination kit or cell number counting with NucleoCounter. Each data point represents the average ± SD of triplicate wells. Similar results were obtained in more than three independent experiments. NS denotes non-significant difference. * P<0.05, ** P<0.01, *** P<0.001.

An MTT cell proliferation assay revealed that 4 nM of RA stimulated proliferation of both murine and human osteoclast progenitors (p<0.001, Figure 2A, B). Cell number count also indicated a similar proliferation pattern in the human CD14^+^ cells treated with 4 nM RA (Figure 2C). Also, initiating osteoclast differentiation by the addition of RANKL did not reduce the proliferation induction capacity of RA. RANKL alone slightly decreased proliferation in RAW 264.7 cells (p<0.05) but had no effect on the human cells. We observed similar proliferation patterns in both murine and human osteoclast progenitors in the MTT assay, when the cells were treated with 400 nM of RA (data no shown). No apparent toxic effect was detected after RA treatment in doses up to 1×10^−6^ M in RAW264.7 and human CD14^+^ cells (data not shown).

### RA does not inhibit mature osteoclast function

**Figure 3:**
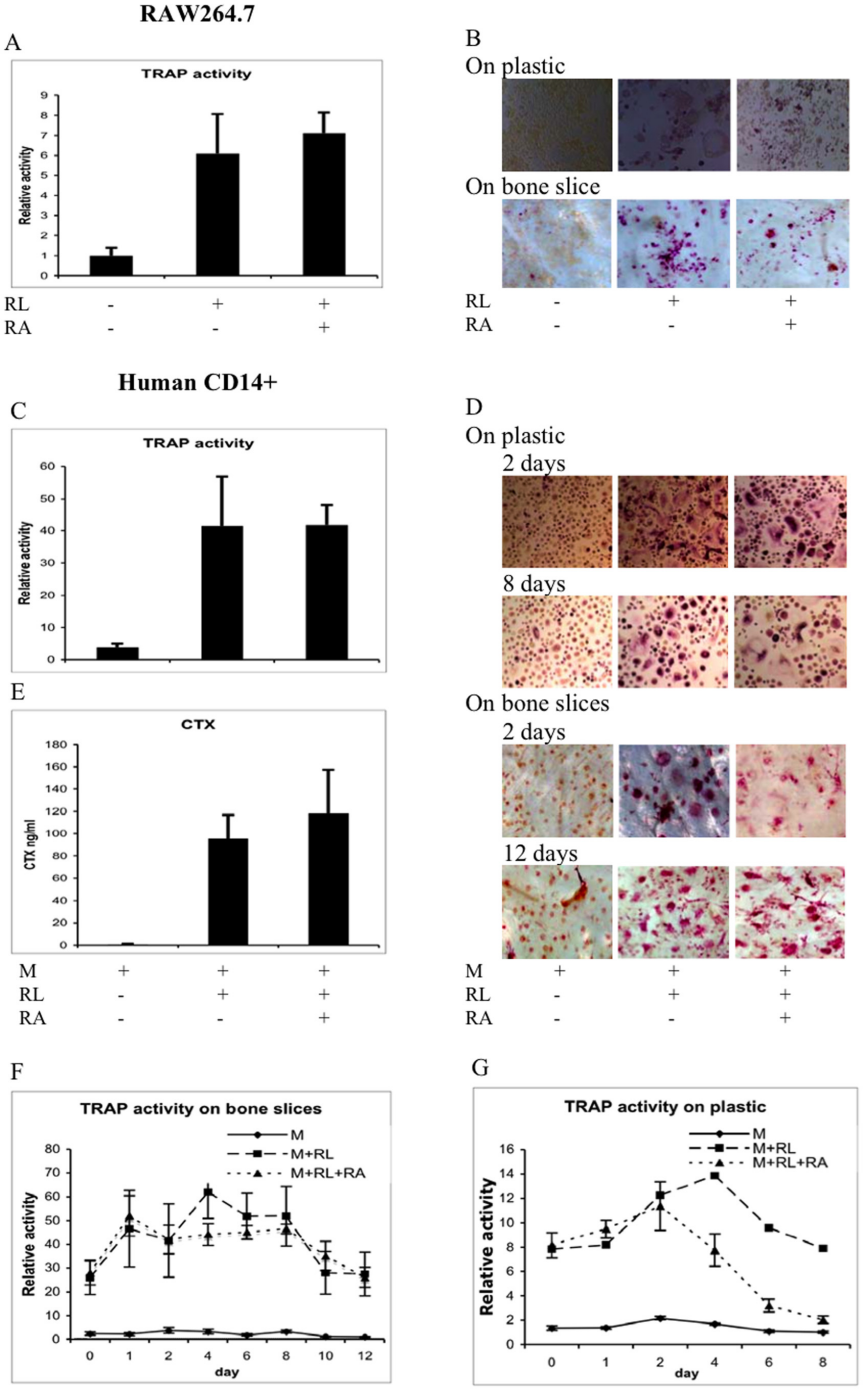
RA does not inhibit mature osteoclast function. RAW264.7 cells were incubated with RANKL on bone slices or plastic for 5 days followed by incubation in 400 nM of RA for another 2 days (A–B). Human CD14^+^ cells were incubated on bone slices or plastic with M-CSF (M) and RANKL (RL) for 14 days or 10 days. The cells were then incubated in 400 nM of RA for another 2 days (D–E), or 12 days (D, F) on bone slices or on plastic for 2 days (C, D) or 8 days (G). The CTX measurements (E), secretion of TRAP activity (A, C, F, G) and the TRAP staining (B, D) were carried out as explained in Figure 1.

The normal physiological level of circulating RA in humans is estimated to be ≤10 nM and effective pharmacological concentration is >100 nM [38], [39]. The highest concentration of RA measured in human plasma upon RA treatment has been shown to be approximately 400 nM [40]. Here when mature murine or human osteoclasts were treated with RA at the highest pharmacological dose of 400 nM for 2 days, no difference was observed in terms of released TRAP activity or cellular TRAP staining (Figure 3A–D). To directly test if these levels of RA affected the ability of human osteoclasts to degrade bones, we measured CTX fragment released into culture media from osteoclasts cultured on bone slices. This revealed that, similar to released TRAP activity, 400 nM of RA for 2 days did not affect CTX fragment release (Figure 3E). In fact human osteoclasts cultured on bone slices, with or without 400 nM RA, show similar stable release of TRAP activity and cellular TRAP staining over a 12-day period (Figure 3D, F). However, if cultured on plastic, osteoclasts receiving 400 nM RA release less TRAP activity at treatment day 4, and by day 8 it is essentially at levels similar to precursor cells not receiving RANKL, although cellular TRAP staining was unaltered (Figure 3D, G).

### RA suppresses RANK expression

**Figure 4:**
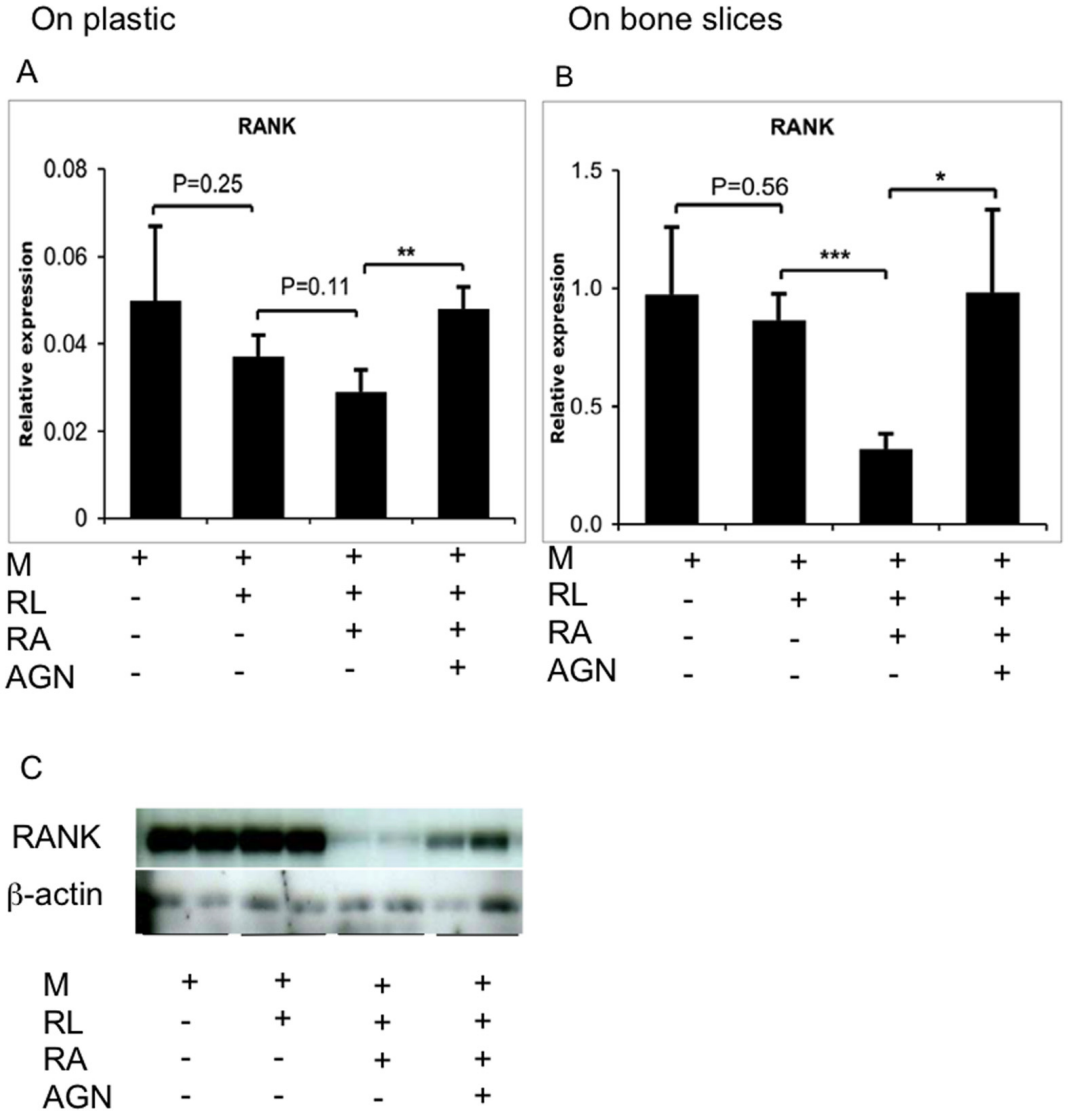
RA inhibits RANK expression in human osteoclast progenitors via RARs. Human CD14^+^ cells were incubated with or without M-CSF, RANKL, RA (4 nM) or AGN (8 nM) on bone slices (B) or plastic (A) for 3 days. mRNA levels were measured using real-time PCR and were normalized relative to the expression of β-actin. (C) Whole cell lysates were subjected to SDS-PAGE and immunoblotted with RANK and β-actin antibodies. * P<0.05, ** P<0.01, *** P<0.001.

The potent inhibitory effect of RA on osteoclast formation suggests that it affects early signaling. Here, as signaling through RANK in preosteoclasts is the key initial osteoclast differentiation factor, we investigated how RA affects RANK expression. Real-time PCR analysis showed that 4 nM of RA potently reduced RANK mRNA expression in differentiation cultures on bone slices on day 3 although the effect was less obvious if cells were cultured on plastic (Figure 4A, B). More importantly, 4 nM of RA almost completely abolished RANK protein production in human osteoclast progenitors (Figure 4C). RANK mRNA expression was fully reversed by 8 nM of the RAR pan-antagonist and protein expression was partially reversed (Figure 4A–C).

### Effects of RA on osteoclast gene expression

**Figure 5:**
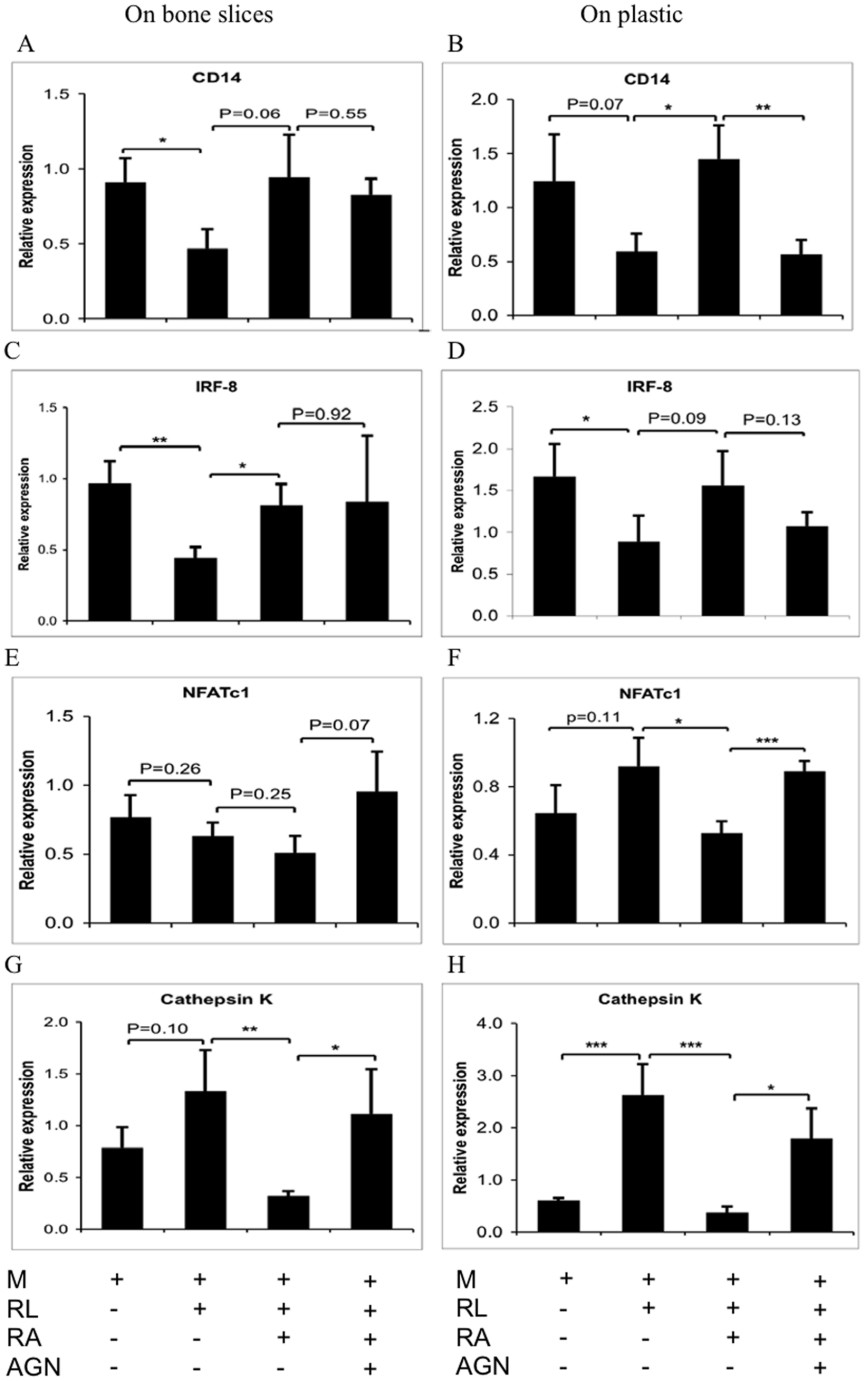
Effects RA on human osteoclast gene expression. Human CD14^+^ cells were treated on bone slices (A, C, E, G) or plastic (B, D, F, H) as described in Figure 4. mRNA levels were measured using real-time PCR and were normalized relative to the expression of β-actin. * P<0.05, ** P<0.01, *** P<0.001.

Finally, we tested how RA affects a set of osteoclast-associated genes during early (day 3) osteoclast differentiation. The progenitor marker CD14 and the negative osteoclastogenic regulator IFR-8 expression were reduced by RANKL (Figure 5A–D). This reduction was prevented by addition of 4 nM of RA. However, whereas 8 nM of the RAR pan-antagonist fully reversed this effect in osteoclast cultured on plastic (Figure 5B, D), no effect was observed on bone cultures (Figure 5A, C). The expression of the positive markers for osteoclasts, NFATc1 and cathepsin K, were induced at a higher level by RANKL in cultures on plastic than the bone (Figure 5E–H). The expression of both genes was reduced by RA via RARs although the result from NFATc1 was less distinct.

## Discussion

This study showed that RA at doses as low as 4 nM significantly repressed RANKL-induced differentiation of osteoclasts from human and murine precursor cells *in vitro*. Our finding is in agreement with previous studies that suggest direct inhibitory effects of RA on osteoclast formation *in vitro* from various mouse tissue sources [25], [32], [41], [42]. The negative effect on osteoclast differentiation by RA could partly be rescued by a RAR pan-antagonist. Therefore, it is reasonable to conclude that the inhibition of osteoclast formation was, at least in part, mediated via RARs. In order to confirm that the effect of RA on osteoclastogenesis in RAW264.7 cells and human CD14^+^ cells were not due to toxic effects of the reagents, cell growth determination assay was performed. RA-induced inhibition of osteoclast formation occurred with no morphological signs of cytotoxicity or decreased cell numbers in either the RAW264.7 or human CD14^+^ cells. On the contrary, cell proliferation was observed in the presence of RA. Moreover, the osteoclast differentiation inducer RANKL did not alter the increased proliferation levels obtained with RA. These findings are in agreement with previous studies that have demonstrated enhancement of proliferation capacity of RA in many types of cells, e.g. chicken macrophages, human giant cell tumors from bone and mouse skin epidermal cells [29], [43], [44]. By contrast, a recent study was unable to show increased proliferation of mouse bone marrow macrophages (BMM) with RA [32]. This discrepancy may be due to the fact that their protocol involved shorter culture periods and higher concentrations of RA. The explanation could also be that there are differences between human CD14^+^ cells and mouse BMM.

Doses of RA (400 nM) for 2 days in culture did not affect the function of mature murine osteoclasts or mature human osteoclasts measured as release of TRAP activity compared to untreated controls. Importantly, 400 nM of RA over 2 days did not affect the bone resorbing activity of mature human osteoclasts as measured by unaltered CTX release. In mature human osteoclasts cultured on bone slices, no difference in released TRAP activity was observed compared with mature human osteoclast controls, even when the cells were treated with 400 nM of RA for 12 days. However, in the plastic culture system an inhibition was observed after 4 days with RA treatment. Therefore, different results may be obtained depending on the culture system used and the length of incubation. This is a novel finding. We believe that osteoclasts cultured on bone slices should reflect the real physiological situation more closely, but the different results might be due to the interactions between the osteoclasts and the micro-environment on bone slices. For instance, an indirect effect via osteoblasts on osteoclasts cannot be excluded since we have previously shown that RA markedly increases the RANKL/OPG ratio in human osteoblasts, which would lead to a stimulation of bone resorption [27]. That mature osteoclasts are activated in the presence of high RA concentrations is in line with the observations of increased bone resorption during hypervitaminosis A and increased bone degradation in organ cultures by retinoids [24], [45]. Also, a few studies using avian and rabbit osteoclasts have shown that RA or retinol could enhance the resorption activity of mature osteoclasts *in vitro* [21], [22], [26]. Our finding that RA exhibits major effects on precursors but little effect on mature osteoclasts is consistent with the recent observation by Conaway et al [32] that an inhibitory effect of RA on osteoclast differentiation in cultured bone marrow macrophages is seen when RA is added at a very early stage but the effect is gradually lost when RA is added later [32].

Our study revealed small but distinct differences in gene expression patterns of the CD14^+^ human osteoclast progenitor cells depending on the carriers for the cell culture. An explanation for this may be provided by the fact that we noticed, upon seeding of osteoclast progenitors, that fewer cells attached to bone slices compared to plastic surfaces, even though a cell density three times that of plastic cultures was seeded on bone slices. That's why we observed a delay in osteoclast formation by 5 to 6 days with the bone slices. Thus, we speculate that the lower initial cell density on bone slices in this study may perhaps cause a slower osteoclast differentiation rate compared to the cultures on plastic. An apparent delay would fit our observation that cultures on plastic show a clearer trend towards induction of the positive regulators of osteoclastogenesis, NFATc1 and cathepsin K, compared to cultures on the bone. NFATc1 is believed to be a master transcription factor for murine osteoclastogenesis [46], [47]. Here we noticed that the induction pattern of cathepsin K expression by human osteoclasts appears earlier compared to NFATc1, which is in agreement with earlier observations in human osteoclast cells [48]. Furthermore RANK mRNA expression appears to be transiently increased by RANKL in mouse BMM cultures on plastic with a peak induction after 2 days followed by reversal to almost control levels after 3 days [32]. Here, cultures on both bone and plastic present no increase in RANK mRNA expression after 3 days of RANKL treatment. However, importantly RA treatment induced a clear RAR-dependent reduction of RANK mRNA in cultures on the bone. The trend was also true for the cultures on plastic although the RA-induced reduction of RANK transcripts did not reach significance. RANK is the key factor present on precursor cells essential for initiating osteoclast differentiation. Here we show that 4 nM of RA almost completely abolished the RANK protein produced by precursor cells and that this was, at least in part, reversible with a RAR pan-antagonist. It has previously been shown that TGF-β, Toll-like receptor agonists and IFN-γ reduce RANK protein expression on human osteoclast precursors. Our study shows that also RA is a potent inhibitor of RANK protein expression in these cells [49], [50].

IRF-8 has been shown to be a negative regulator of osteoclastogenesis. Mice deficient in IFR-8 show severe osteoporosis, owing to increased numbers of osteoclasts [20]. In human CD14^+^ cells, RANKL overcomes this negative regulation and stimulates osteoclast formation by decreasing expression of IRF-8 in cells cultured on both bone slices and plastic. In the present study, RA antagonized the RANKL-induced reduction of IRF-8, which is in line with the finding that RA inhibits the expression of the RANKL receptor and thereby makes precursor cells refractory to the effects of RANKL. IFR-8 is thought to function as a negative regulator of osteoclast formation by binding NFATc1 and preventing transactivation of NFATc1. This possibly explains why RA also suppressed NFATc1 in our cultures. We did not detect any effect of RA on the expression of MafB, another repressor of osteoclastogenesis, in our primary human cultures as was recently shown for murine cells [32].

In conclusion, our results suggest that the major impact of RA on osteoclast biology *in vitro* is its substantial effects on precursor cells. RA increases osteoclast precursor proliferation and potently inhibits RANKL-stimulated osteoclast differentiation in both human and murine cells. An RA concentration as low as 4 nM suppresses RANK mRNA and protein expression in human osteoclast progenitors. The strong capacity of RA to suppress the early key osteoclastogenesis factor, the RANK protein, may explain its potent ability to inhibit osteoclastogenesis.

## Supporting Information

Figure S1 RA inhibits differentiation of osteoclast progenitor human CD14+ cells. Number of multinucleated TRAP-positive cells in human CD14+ cells incubated with M-CSF (M, 25 ng/ml) and RANKL (RL, 25 ng/ml) with various concentrations of RA on plastic for 10 days was counted. The TRAP staining was carried out as described in Figure 1. Each data point represents the average ± SD of triplicate wells. Similar results were obtained in more than three independent experiments. *** P<0.001, compared with RL group.
(6.02 MB TIF)

Figure S2 Expanded Figure 1C, 1F. The TRAP staining was carried out as explained in Figure 1.
(6.02 MB TIF)
