# Supplementary figures and images for: Retinoic Acid Increases Proliferation of Human Osteoclast Progenitors and Inhibits RANKL-Stimulated Osteoclast Differentiation by Suppressing RANK
Source: PLoS One. 2010 Oct 11;5(10):e13305. doi: 10.1371/journal.pone.0013305 (PMC2952600; doi:10.1371/journal.pone.0013305)

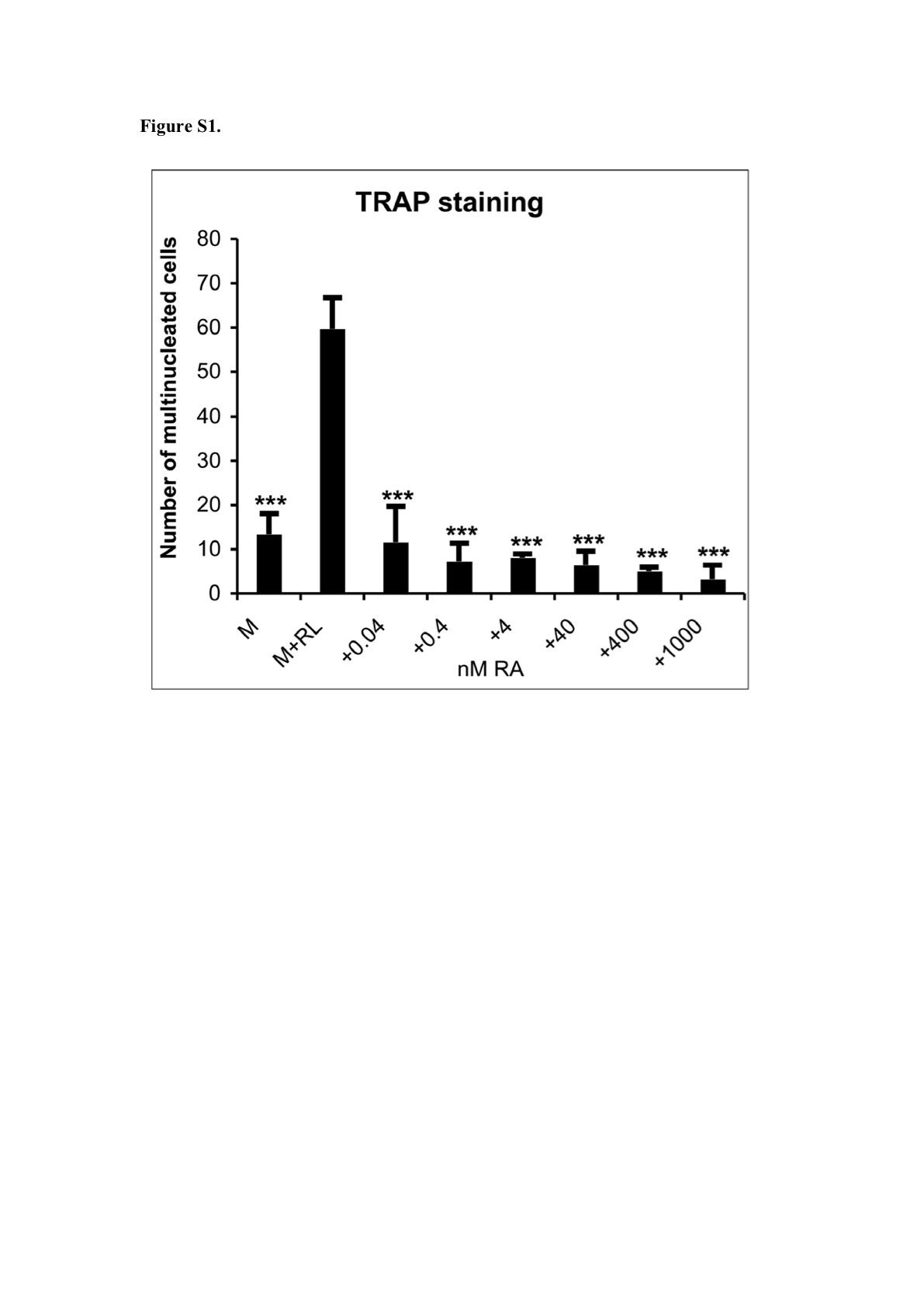

Supplement: Figure S1 — RA inhibits differentiation of osteoclast progenitor human CD14+ cells. Number of multinucleated TRAP-positive cells in human CD14+ cells incubated with M-CSF (M, 25 ng/ml) and RANKL (RL, 25 ng/ml) with various concentrations of RA on plastic for 10 days was counted. The TRAP staining was carried out as described in Figure 1. Each data point represents the average ± SD of triplicate wells. Similar results were obtained in more than three independent experiments. *** P<0.001, compared with RL group. (6.02 MB TIF) [file pone.0013305.s001.tif]

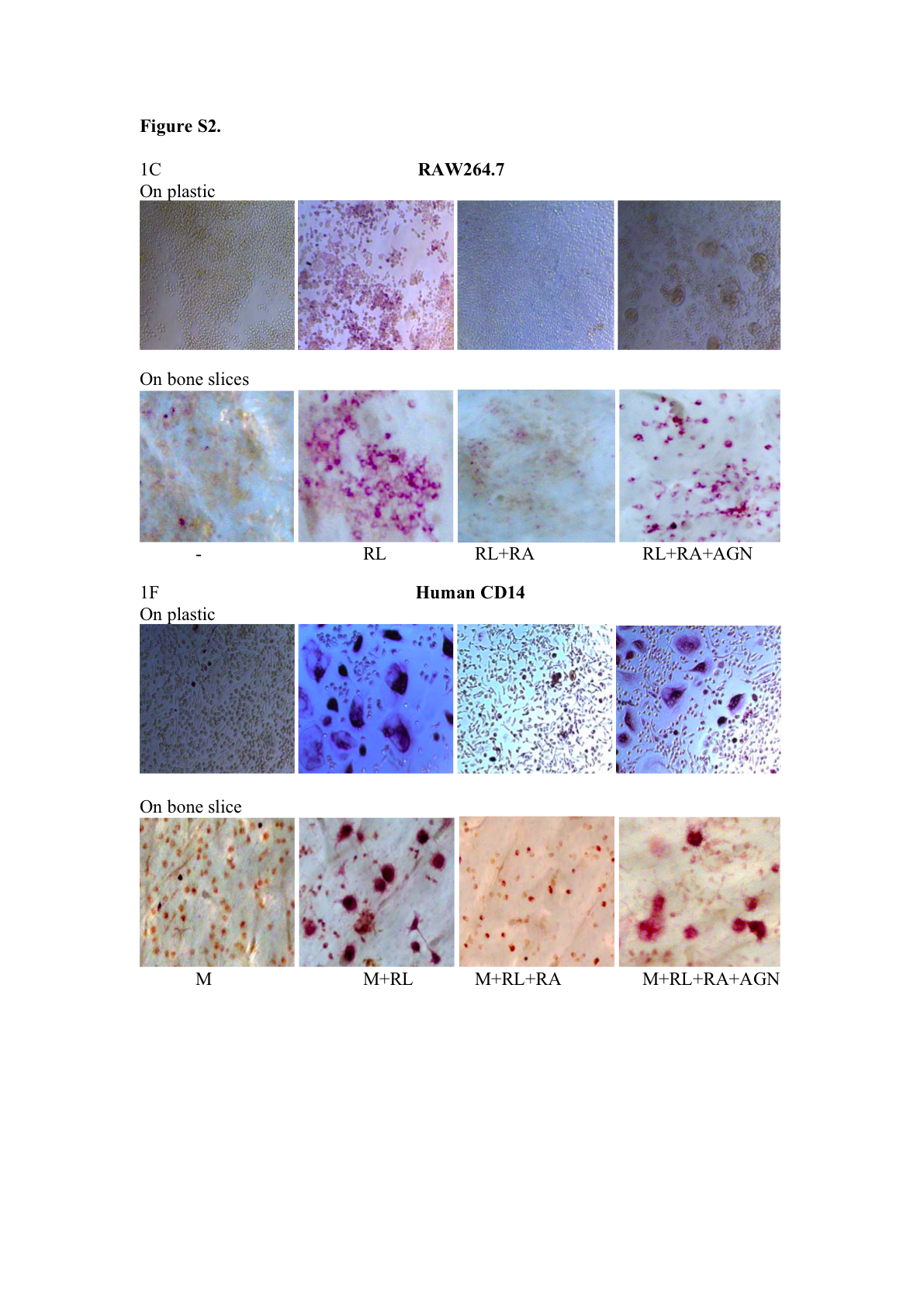

Supplement: Figure S2 — Expanded Figure 1C, 1F. The TRAP staining was carried out as explained in Figure 1. (6.02 MB TIF) [file pone.0013305.s002.tif]
